# Supplementary material for: Developing an Anatomically Valid Segmentation Protocol for Anterior Regions of the Medial Temporal Lobe for Neurodegenerative Diseases
Source: Hippocampus. 2025 Jul 30;35(5):e70027. doi: 10.1002/hipo.70027 (PMC12361719; doi:10.1002/hipo.70027)
Supplement: Supplementary file 1 — Data S1. hipo70027‐sup‐0001‐Supinfo. [file HIPO-35-0-s001.docx]

**Supplementary Methods:
Criteria for medial temporal lobe subregion annotations on histological sections**The annotations of MTL cortical regions on Nissl stained sections followed previously described cytoarchitectonic criteria (ERC: (1–10); BA35: (1,10–19); BA36: (11–19)).

**Supplementary results:**

**Supplementary Table 1*.*** Demographic and diagnostic details for subjects included in this study.

Abbreviations: ADNC: Alzheimer’s disease neuropathologic change; CVD: cerebrovascular disease; CAA: cerebral amyloid angiopathy; CBD: corticobasal degeneration; CNDR: Center for Neurodegenerative Disease Research; HNL/UCLM: the Human Neuroanatomy Lab at the University of Castilla-La Mancha; LBD: Lewy body disease; FTLD-PPA (PNFA): frontotemporal lobar degeneration-primary progressive aphasia (progressive nonfluent aphasia); PD: Parkinson’s disease; PSP: progressive supranuclear palsy; PART: primary age-related tauopathy; FTLD-bvFTD: frontotemporal lobar degeneration-behavioral variant frontotemporal dementia; FTLD-TDP: frontotemporal lobar degeneration with TDP-43 inclusions.

| **Patient ID** | **Hemisphere** | **Age (years)** | **Sex** | **Clinical Diagnosis** | **Neuropathological Diagnosis** | **PMI (hours)** |
| --- | --- | --- | --- | --- | --- | --- |
| HNL/UCLM-01 | L | 78 | F | Unknown | Low ADNC, CVD | 6 |
| HNL/UCLM-02 | R | 90+ | M | Unknown | Intermediate ADNC, CAA | 11 |
| HNL/UCLM-03 | R | 74 | M | Unknown | Pathological aging (mild tau pathology, B2), and mild CAA | 3 |
| HNL/UCLM-04 | R | 61 | M | Unknown | Pathological aging (mild tau pathology, B1), suspected CBD | 16 |
| HNL/UCLM-05 | R | 76 | F | Unknown | No pathology | 4 |
| HNL/UCLM-06 | L | 66 | F | Unknown | No pathology, Incidental LBD | 9 |
| HNL/UCLM-07 | R | 90+ | F | Unknown | Low ADNC | 16 |
| HNL/UCLM-08 | L | 90 | M | Unknown | Low ADNC, Brainstem predominant Incidental LBD | 7 |
| HNL/UCLM-09 | R | 74 | M | Unknown | Low ADNC | 2 |
| HNL/UCLM-10 | L | 62 | F | Unknown | Low ADNC | 3 |
| CNDR-01 | R | 76 | F | FTLD-PPA (PNFA) | 1.CBD 2. low ADNC | 14 |
| CNDR-02 | R | 90+ | M | PD with Dementia | 1.LBD, 2. high ADNC | 17 |
| CNDR-03 | L | 86 | M | Probable AD | 1. high ADNC ,2. LATE | 10 |
| CNDR-04 | R | 90+ | F | Normal | 1. intermediate ADNC, 2. LBD | 6 |
| CNDR-05 | L | 79 | F | PSP | 1. PSP, 2. PART | 5 |
| CNDR-06 | R | 70 | M | PD with Dementia | 1. LBD, 2. PART | 9.5 |
| CNDR-07 | L | 80 | M | FTLD-bvFTD | 1.Argyrophilic grain disease, 2.PSP | 28 |
| CNDR-08 | R | 77 | M | Corticobasal syndrome | 1. CBD, 2. FTLD-TDP | 4 |
| CNDR-09 | R | 82 | F | Dementia of undetermined etiology | 1.FTLD-TDP, 2.PART | 12 |
| CNDR-10 | R | 83 | R | Corticobasal syndrome | 1. PSP, 2. low ADNC | 4 |

**Supplementary Figure 1.** Candidate landmarks considered for the development of the segmentation rule*s* for the anterior borders of the MTL cortical subregions*.* In each row, the left image shows a slide before the landmark appears, the middle image marks the first slide where the landmark appears, and the right image shows a subsequent slide.
Please note that panel (a) includes three slides showing the transition of the temporal pole, and the left–middle–right structure described above does not apply to this panel.

Panels (a–e) depict the appearance of specific anatomical landmarks: (a) temporal pole, (b) limen insulae, (c) amygdala, (d) hippocampus, and (e) collateral sulcus. These landmarks were selected because they can be observed in both histology and MRI and help anchor segmentation rules across modalities.


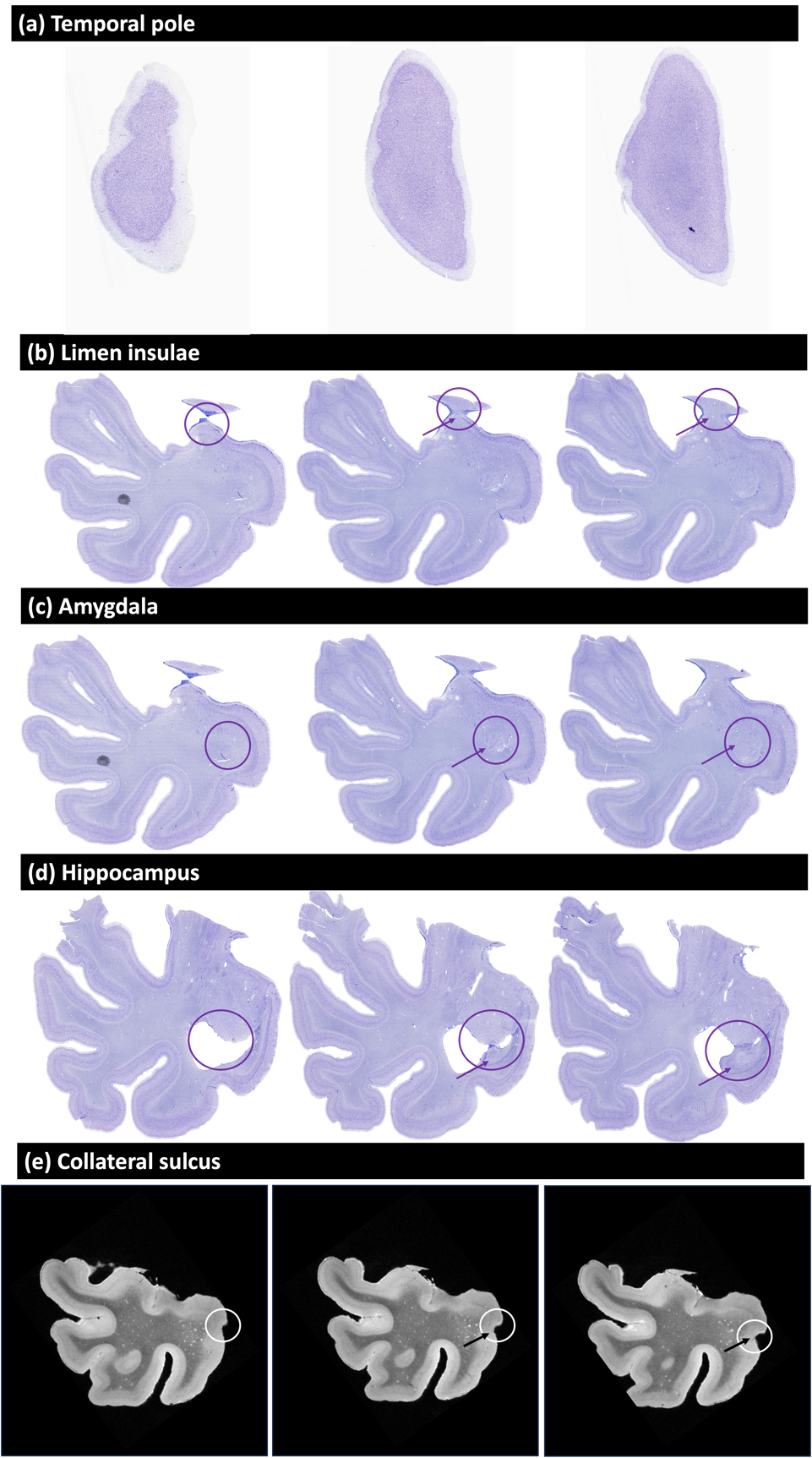


**Supplementary Figure 2.** The measurement of distances on the histology sections using Adobe Illustrator. Black lines are neuroanatomist’s annotations of boundaries between MTL subregions, and the red curve measures the distance between a given boundary (BA35 lateral border) and a given anatomical landmark proposed for consideration in the MRI protocol (fundus of the CS).
Note that histology annotations shown here include fine-grained partitions of BA35 into subregions 35v, 35o, and 35d. These were combined as just BA35 for the development of the protocol.

By measuring the length of the scale bar in Adobe Illustrator units, we were able to convert all the measurements to units of millimeters. In the image, the 5 mm scale bar corresponds to ∼27.5 mm in Adobe Illustrator. Hence the length of the red curve between the CS fundus and lateral border of the BA35 is estimated to be 3.4 mm.


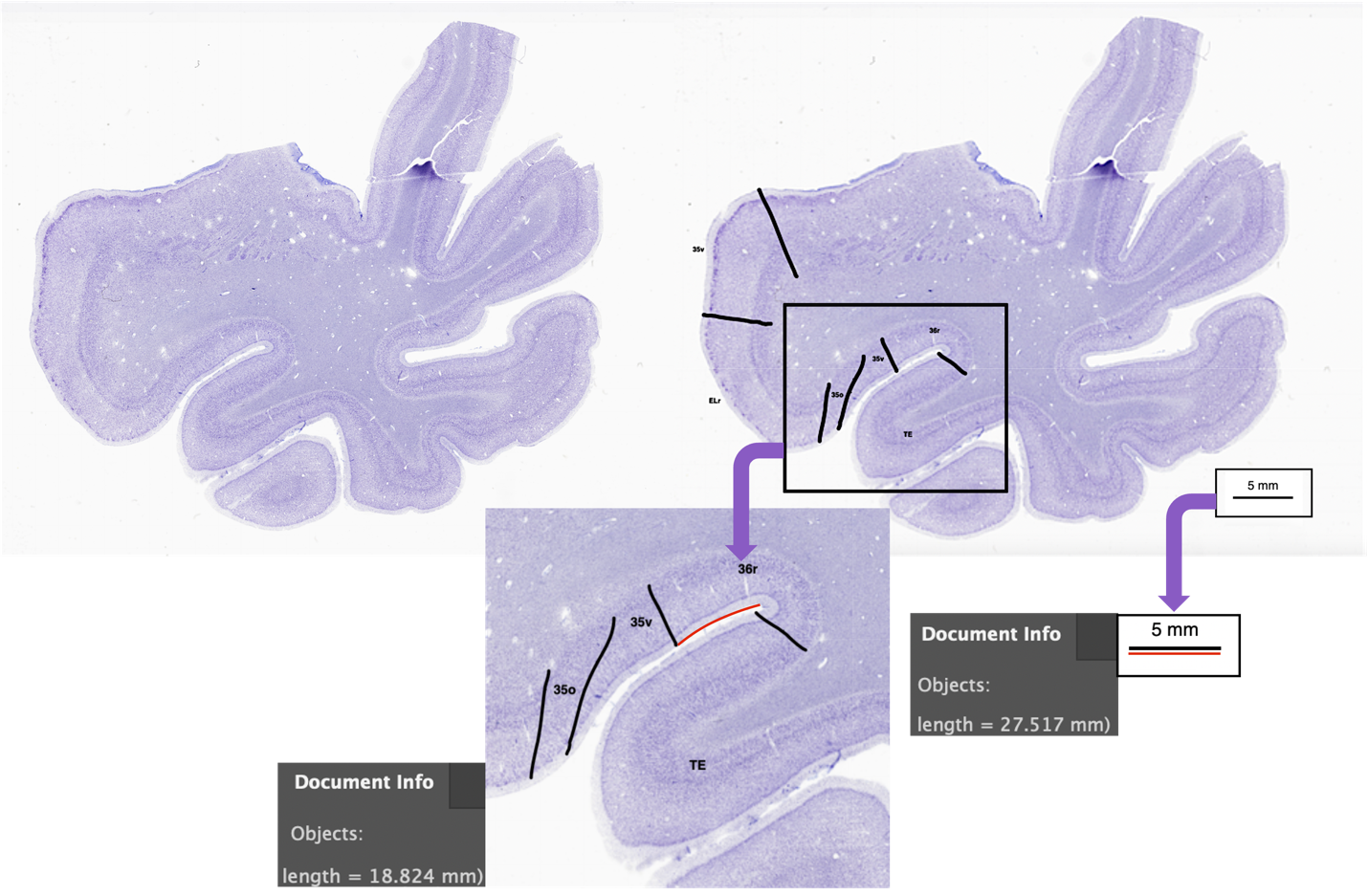


**Supplementary Figure 3.** Landmarks observable on MRI in coronal slices. Abbreviations: PHG: parahippocampal gyrus; FG: fusiform gyrus; CS: collateral sulcus

**
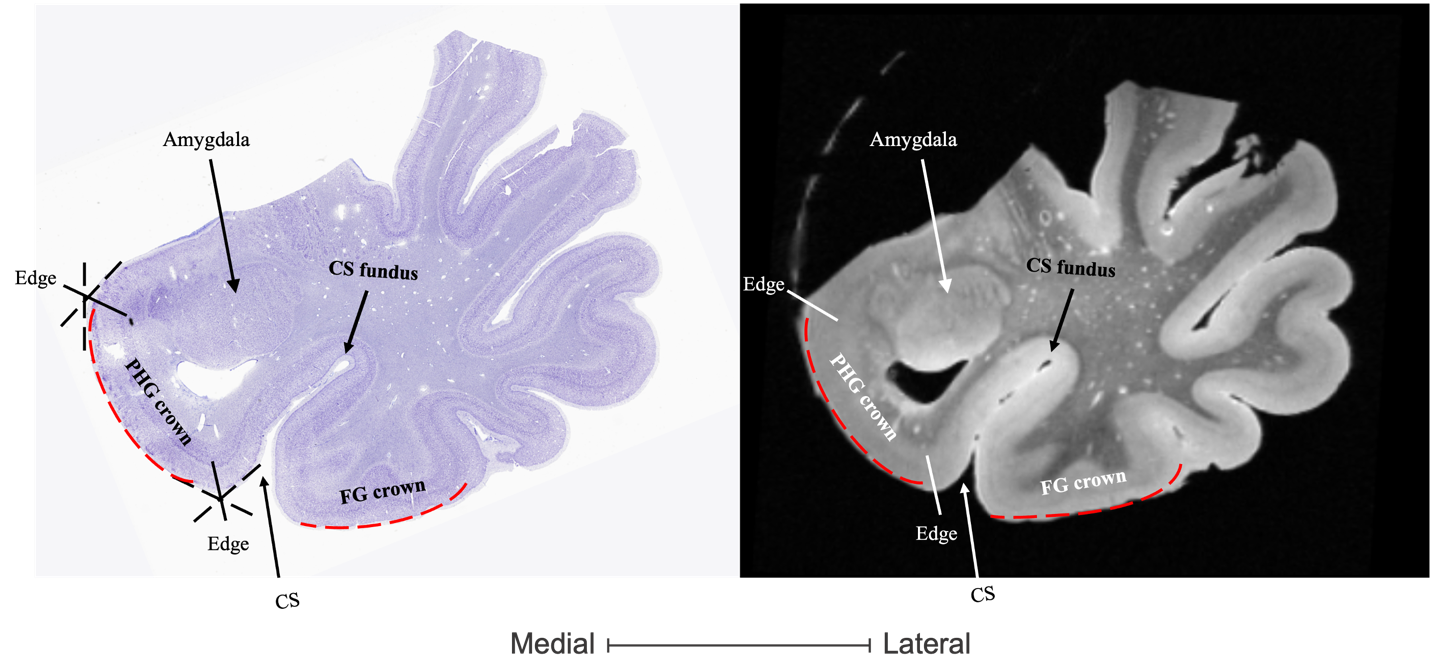
**

**Supplementary Table 2.** Summary of the composition of the in vivo atlas set, including demographics, and cognitive testing. The P‐values are two‐tailed and computed using the t‐test for numerical variables and using the Fisher exact test for sex. Abbreviations: MMSE: Mini‐Mental State Examination; CERAD: Consortium to Establish a Registry for Alzheimer's Disease.

|  | **Atlas subset (*n* = 29)** | | | |  |
| --- | --- | --- | --- | --- | --- |
|  | **NC (*n* = 15)** | | **aMCI (*n* = 14)** | |  |
|  | **Mean ± S.D.** | **Range** | **Mean ± S.D.** | **Range** | ***P*** |
| Sex (male/female) | 7/8 | | 6/8 | | 1.0000 |
| Age | 66.3 ± 9.5 | 54−84 | 71.9 ± 6.2 | 63−80 | 0.0696 |
| Education  (years) | 15.6 ± 2.6 | 12−20 | 16.9 ± 2.8 | 12−20 | 0.1994 |
| MMSE | 29.5 ± 1.0 | 27−30 | 26.9 ± 1.7 | 24−30 | 0.0001 |
| CERAD word list total | 24.7 ± 2.9 | 21−29 | 16.2 ± 3.2 | 11−23 | 0.0000 |
| Delayed recall | 8.7 ± 1.8 | 4−10 | 3.4 ± 2.1 | 0−8 | 0.0000 |

**Supplementary Figure 4.** Measured distances from the cytoarchitectonic borders of ERC to the chosen landmarks visualized in violin plots. This is a visualization of Table 3 in the main manuscript*.*

For all borders, a negative value reflects the situation where the actual border is located medial of the chosen landmark and a positive value reflects the situation where the actual border is located lateral to the chosen landmark.

Abbreviations: ERC: entorhinal cortex; CS: collateral sulcus; PHG: parahippocampal gyrus; SD: standard deviation; H: hippocampus

**
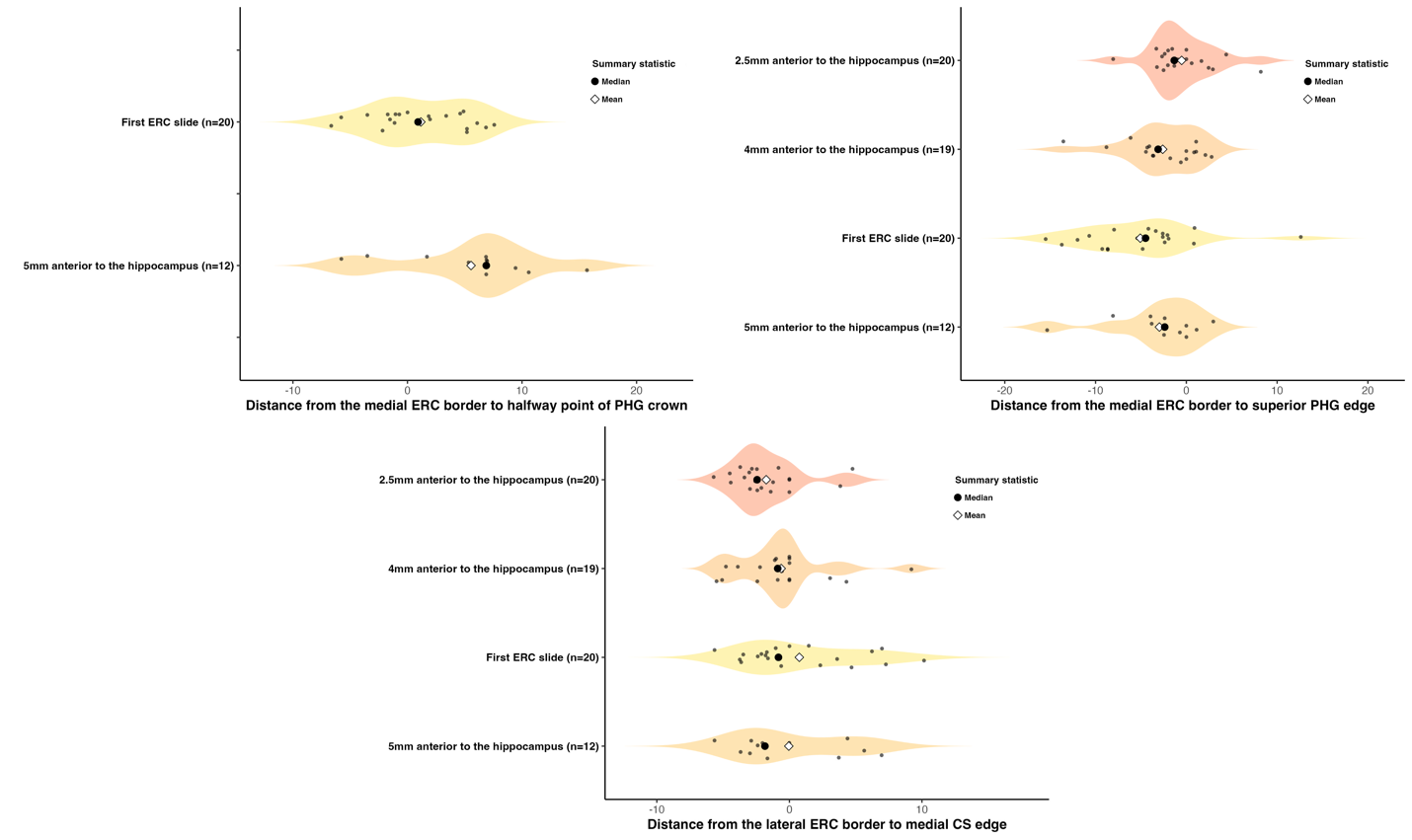
**

**Supplementary Figure 5.** Measured distances from the cytoarchitectonic borders of BA35 to the chosen landmarks visualized in violin plots. This is a visualization of Table 4 in the main manuscript.

For all borders, a negative value reflects the situation where the actual border is located medial of the chosen landmark and a positive value reflects the situation where the actual border is located lateral to the chosen landmark.

Abbreviations: BA35: Brodmann area 35; CS: collateral sulcus; PHG: parahippocampal gyrus; SD: standard deviation; H: hippocampus.


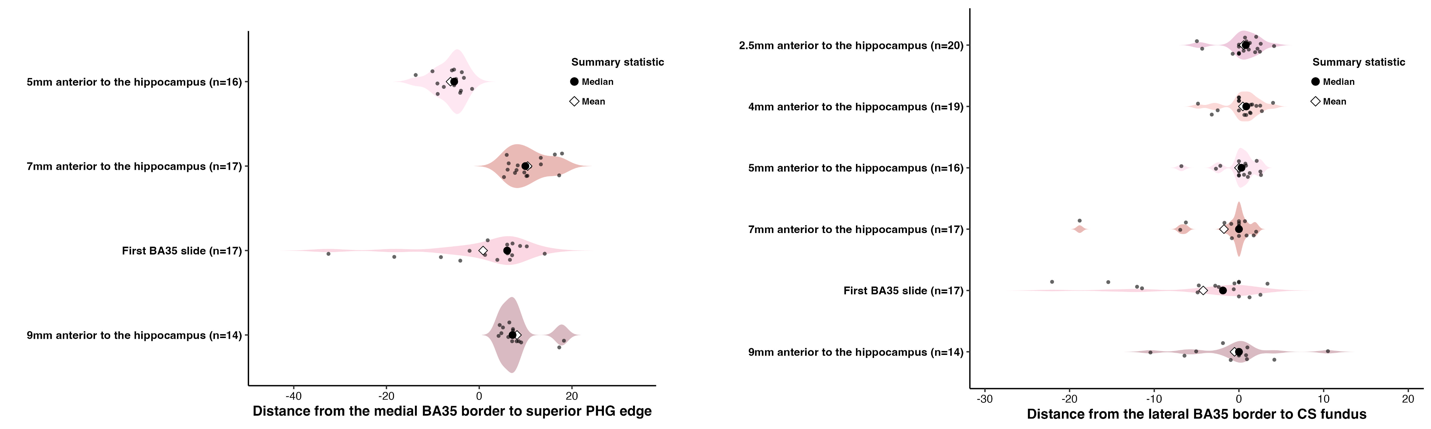


**Supplementary Figure 6.** Measured distances from the cytoarchitectonic borders of BA36 to the chosen landmarks visualized in violin plots. This is a visualization of Table 5 in the main manuscript. For all borders, a negative value reflects the situation where the actual border is located to the medial of the chosen landmark and a positive value reflects the situation where the actual border is located lateral to the chosen landmark.

Abbreviations: BA36: Brodmann area 36; CS: collateral sulcus; SD: standard deviation; H: hippocampus; FG: fusiform gyrus.


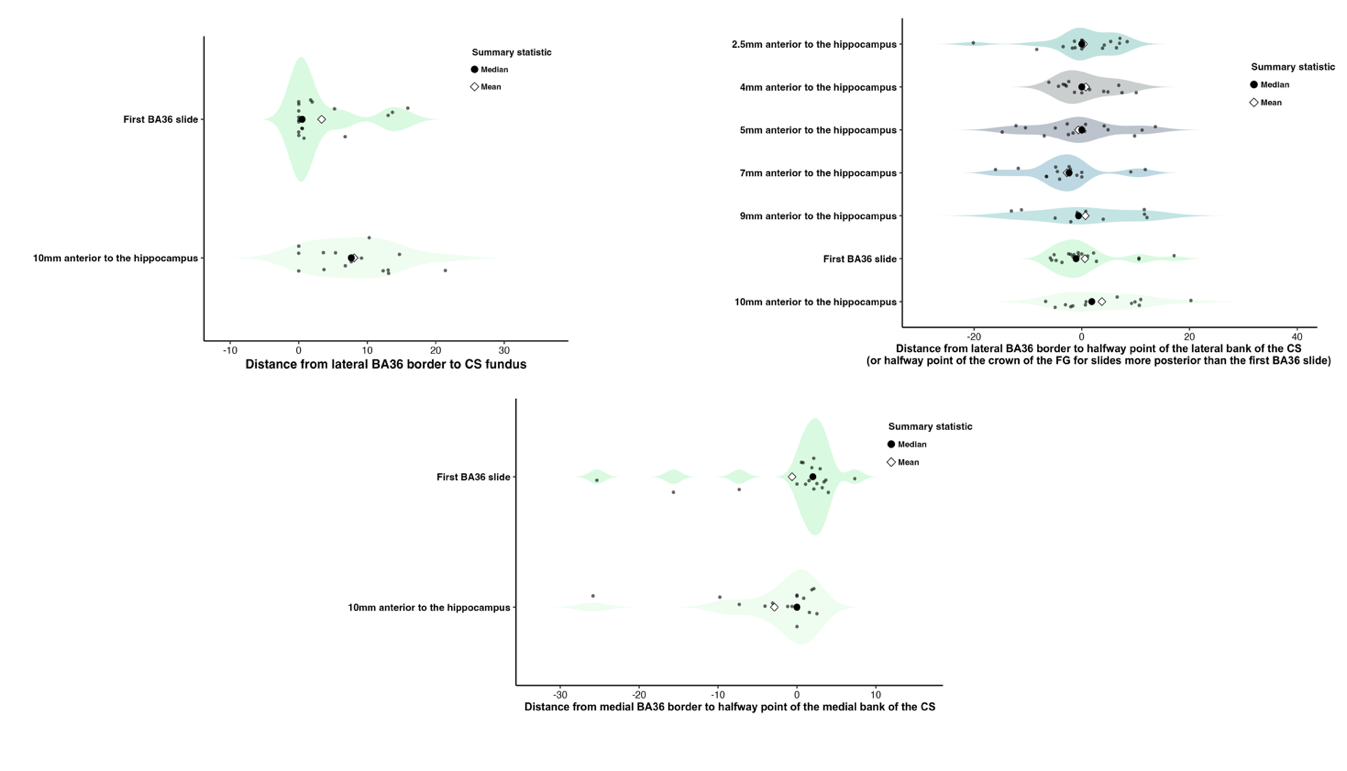


**Supplementary Table 3.** Distances from the ERC histological boundaries to landmarks observable on MRI for cases with a deep vs. shallow collateral sulcus and cases with neurodegenerative diseases vs. without neurodegenerative diseases. A negative value reflects the situation where the actual border is located laterally of the landmark and a positive value where the actual border is located medially of the landmark. Abbreviations: CS: collateral sulcus; PHG: parahippocampal gyrus; NDD: neurodegenerative disease

Please note that when comparing cases with deep vs. shallow CS, we only considered borders in the vicinity of the CS to show consistency of the border placement between the two groups in this area.
*The borders outside CS area were marked with N/A.
A cut-off of 7mm in the first slide where the hippocampal head appears was used to determine if cases had a shallow or deep CS(20).

| **ERC** | | | | | | | | | |
| --- | --- | --- | --- | --- | --- | --- | --- | --- | --- |
| **5mm anterior to the hippocampus** | | | | | | | | | |
|  | **Lateral ERC border to** | | | **Medial ERC border to** | | | | | |
|  | **Medial CS edge** | | | **Superior PHG edge** | | | **Halfway point of PHG crown** | | |
| Variable: | Mean | Median | SD | Mean | Median | SD | Mean | Median | SD |
| Deep CS | -0.02 | -1.66 | 4.50 | N/A* | | | | | |
| Shallow CS | -0.09 | -2.03 | 4.10 | N/A* | | | | | |
| With NDD | 0.45 | -1.85 | 4.27 | -2.46 | -0.36 | 5.70 | 7.00 | 6.94 | 6.10 |
| Without NDD | -1.04 | -1.44 | 4.29 | -4.31 | -2.46 | 3.27 | 1.69 | 1.71 | 5.18 |
| **First ERC slide** | | | | | | | | | |
|  | **Lateral ERC border to** | | | **Medial ERC border to** | | | | | |
|  | **Medial CS edge** | | | **Superior PHG edge** | | | **Halfway point of PHG crown** | | |
| Variable: | Mean | Median | SD | Mean | Median | SD | Mean | Median | SD |
| Deep CS | 1.20 | 0.73 | 4.40 | N/A* | | | | | |
| Shallow CS | 0.30 | -1.75 | 4.58 | N/A* | | | | | |
| With NDD | 0.85 | 0.00 | 4.34 | -5.71 | -4.81 | 5.12 | 2.16 | 3.38 | 4.12 |
| Without NDD | 0.63 | -1.03 | 4.72 | -4.30 | -2.60 | 7.65 | -0.05 | -1.04 | 4.20 |
| **4mm anterior to the hippocampus** | | | | | | | | | |
|  | **Lateral ERC border to** | | | **Medial ERC border to** | | | | | |
|  | **Medial CS edge** | | | **Superior PHG edge** | | | **Halfway point of PHG crown** | | |
| Variable: | Mean | Median | SD | Mean | Median | SD | Mean | Median | SD |
| Deep CS | -0.96 | -0.28 | 3.32 | N/A* | | |  |  |  |
| Shallow CS | -0.25 | -0.93 | 3.76 | N/A* | | |  |  |  |
| With NDD | -1.83 | -0.91 | 2.03 | -1.83 | -1.56 | 3.56 |  |  |  |
| Without NDD | 0.73 | 0.00 | 4.27 | -3.45 | -3.19 | 4.52 |  |  |  |
| **2.5mm anterior to the hippocampus** | | | | | | | | | |
|  | **Lateral ERC border to** | | | **Medial ERC border to** | | | | | |
|  | **Medial CS edge** | | | **Superior PHG edge** | | | **Halfway point of PHG crown** | | |
| Variable: | Mean | Median | SD | Mean | Median | SD | Mean | Median | SD |
| Deep CS | -0.83 | -1.12 | 3.22 | N/A* | | |  |  |  |
| Shallow CS | -2.67 | -2.78 | 1.36 | N/A* | | |  |  |  |
| With NDD | -1.87 | -2.11 | 1.26 | -0.28 | -0.94 | 4.16 |  |  |  |
| Without NDD | -1.61 | -2.58 | 3.72 | -0.85 | -1.67 | 2.50 |  |  |  |

**Supplementary Table 4.** Distances from BA35 histological boundaries to landmarks observable on MRI for cases with a deep vs. shallow collateral sulcus and cases with neurodegenerative diseases vs. without neurodegenerative diseases. A negative value reflects the situation where the actual border is located laterally of the landmark and a positive value where the actual border is located medially of the landmark.

Abbreviations: CS: collateral sulcus; PHG: parahippocampal gyrus; NDD: neurodegenerative disease

Please note that when comparing cases with deep vs. shallow CS, we only considered borders in the vicinity of the CS to show consistency of the border placement between the two groups in this area.
*The borders beyond CS area were marked with N/A.
A cut-off of 7mm in the first slide where the hippocampal head appears was used to determine if cases had a shallow or deep CS(20).

| **BA35** | | | | | | |
| --- | --- | --- | --- | --- | --- | --- |
| **9mm anterior to the hippocampus** | | | | | | |
|  | **Lateral border of BA35 to** | | | **Medial border of BA35 to** | | |
|  | **CS fundus** | | | **Superior PHG edge** | | |
| variable: | Mean | Median | SD | Mean | Median | SD |
| Deep CS | -1.89 | -0.97 | 4.70 | N/A* | | |
| Shallow CS | -0.77 | 0.00 | 2.81 | N/A* | | |
| With NDD | -0.71 | 0.00 | 5.68 | 9.29 | 8.07 | 5.08 |
| Without NDD | -0.44 | -0.45 | 3.95 | 6.09 | 6.49 | 1.27 |
| **7mm anterior to the hippocampus** | | | | | | |
|  | **Lateral border of BA35 to** | | | **Medial border of BA35 to** | | |
|  | **CS fundus** | | | **Superior PHG edge** | | |
| variable: | Mean | Median | SD | Mean | Median | SD |
| Deep CS | -3.78 | 0.00 | 6.86 | N/A* | | |
| Shallow CS | 0.02 | 0.00 | 1.11 | N/A* | | |
| With NDD | -0.93 | 0.00 | 2.47 | 9.25 | 9.35 | 4.31 |
| Without NDD | -2.72 | 0.00 | 6.95 | 10.48 | 9.96 | 4.18 |
| **5mm anterior to the hippocampus** | | | | | | |
|  | **Lateral border of BA35 to** | | | **Medial border of BA35 to** | | |
|  | **CS fundus** | | | **Superior PHG edge** | | |
| variable: | Mean | Median | SD | Mean | Median | SD |
| Deep CS | -1.11 | 0.00 | 2.77 | N/A* | | |
| Shallow CS | 1.11 | 0.95 | 1.01 | N/A* | | |
| With NDD | 1.12 | 0.60 | 1.24 | N/A* | | |
| Without NDD | -0.87 | 0.00 | 2.64 | N/A* | | |
| **4mm anterior to the hippocampus** | | | | | | |
|  | **Lateral border of BA35 to** | | | **Medial border of BA35** | | |
|  | **CS fundus** | | |  | | |
| Variable: | Mean | Median | SD |  |  |  |
| Deep CS | -0.21 | 0.32 | 2.63 | Lateral border of ERC | | |
| Shallow CS | 1.21 | 1.38 | 1.03 | Lateral border of ERC | | |
| With NDD | 1.31 | 1.12 | 1.32 | Lateral border of ERC | | |
| Without NDD | -0.48 | 0.00 | 2.48 | Lateral border of ERC | | |
| **2.5mm anterior to the hippocampus** | | | | | | |
|  | **Lateral border of BA35 to** | | | **Medial border of BA35** | | |
|  | **CS fundus** | | |  | | |
| Variable: | Mean | Median | SD |  |  |  |
| Deep CS | -0.38 | 0.00 | 2.47 | Lateral border of ERC | | |
| Shallow CS | 1.49 | 1.23 | 1.24 | Lateral border of ERC | | |
| With NDD | 1.27 | 1.16 | 1.01 | Lateral border of ERC | | |
| Without NDD | -0.33 | 0.00 | 2.82 | Lateral border of ERC | | |

**Supplementary Table 5.** Distances from BA36 histological boundaries to landmarks observable on MRI for cases with a deep vs. shallow collateral sulcus and cases with neurodegenerative diseases vs. without neurodegenerative diseases. A negative value reflects the situation where the actual border is located laterally of the landmark and a positive value where the actual border is located medially of the landmark.

Abbreviations: CS: collateral sulcus; PHG: parahippocampal gyrus; NDD: neurodegenerative disease

Please note that when comparing cases with deep vs. shallow CS, we only considered borders in the vicinity of the CS to show consistency of the border placement between the two groups in this area.
*The borders beyond CS area were marked with N/A.
A cut-off of 7mm in the first slide where the hippocampal head appears was used to determine if cases had a shallow or deep CS (20).

| **BA36** | | | | | | |
| --- | --- | --- | --- | --- | --- | --- |
| **10mm anterior to the hippocampus** | | | | | | |
|  | **Lateral border of BA36 to** | | | **Medial border of BA36 to** | | |
|  | **CS fundus** | | | **Halfway point of the medial bank of the CS** | | |
| variable: | Mean | Median | SD | Mean | Median | SD |
| Deep CS | 1.83 | 0.00 | 2.59 | 1.03 | 0.57 | 3.92 |
| Shallow CS | 5.06 | 0.53 | 6.93 | -3.71 | 0.23 | 9.89 |
| With NDD | 2.90 | 0.00 | 5.54 | -0.99 | 0.55 | 6.03 |
| Without NDD | 4.13 | 2.36 | 5.36 | -1.77 | 0.57 | 9.83 |
| **2.5 to 9mm anterior to the hippocampus** | | | | | | |
|  | **Lateral border of BA36 to** | | | **Medial border of BA36** | | |
|  | **Halfway point of PHG crown** | | |  | | |
|  | N/A* | | | Lateral border of BA35 | | |

**Supplementary Table 6.** Volume comparisons of anterior entorhinal cortex (ERC), Brodmann area 35 (BA35), and Brodmann area 36 (BA36) between cases with deep and shallow collateral sulcus (CS). Volumes were obtained using automatic segmentation, and group differences were assessed using a two-sample t-test. No significant differences were observed across the groups (p > 0.05 for all comparisons).

| Region | Shallow sulcus  Volume (mm^3^)  Mean±SD | Deep sulcus  Volume (mm^3^)  Mean±SD | T  statistic | P  value |
| --- | --- | --- | --- | --- |
| ERC | 717.79±77.68 | 719.90±140.25 | 0.04 | 0.96 |
| BA35 | 997.52±185.49 | 1064.04±133.68 | 1.11 | 0.27 |
| BA36 | 2336.33±550.02 | 2300.57±346.56 | -0.21 | 0.83 |

*Abbreviations: ERC=entorhinal cortex; BA=Brodmann area.*

**Supplementary Figure 7.** Modified lateral border of BA36 for the second section of this region located 9 mm anterior to the head of the hippocampus. The yellow lines indicate the medial and lateral borders of BA35 and BA36, according to the protocol. The solid green line reflects the distance of 7 mm from the edge of the collateral sulcus, which was used to place the medial border of BA35. The dashed green line represents the distance from the fundus of the collateral sulcus to the midpoint of the fusiform gyrus; the lateral border of BA36 was placed at the midpoint of this distance to ensure smoother transitions of borders across adjacent sections.
Abbreviations: BA=Brodmann area; CS= collateral sulcus

**Supplementary Figure 8.** 3D rendering of a manual segmentation of one case according to the developed protocol highlighting the consistency of the borders between consecutive slices in a control and MCI case from the in vivo dataset. Case 1 is a control subject and corresponds to Case 1 in Supplementary Figure 6; Case 2 is an individual with mild cognitive impairment (MCI) and corresponds to Case 3 in Supplementary Figure 6. For both cases, the top panel depicts all three labels, while the lower two panels focus on the border between either ERC and BA35 or between BA35 and BA36.

Abbreviations: BA=Brodmann area; ERC=entorhinal cortex; MCI: mild cognitive impairment.


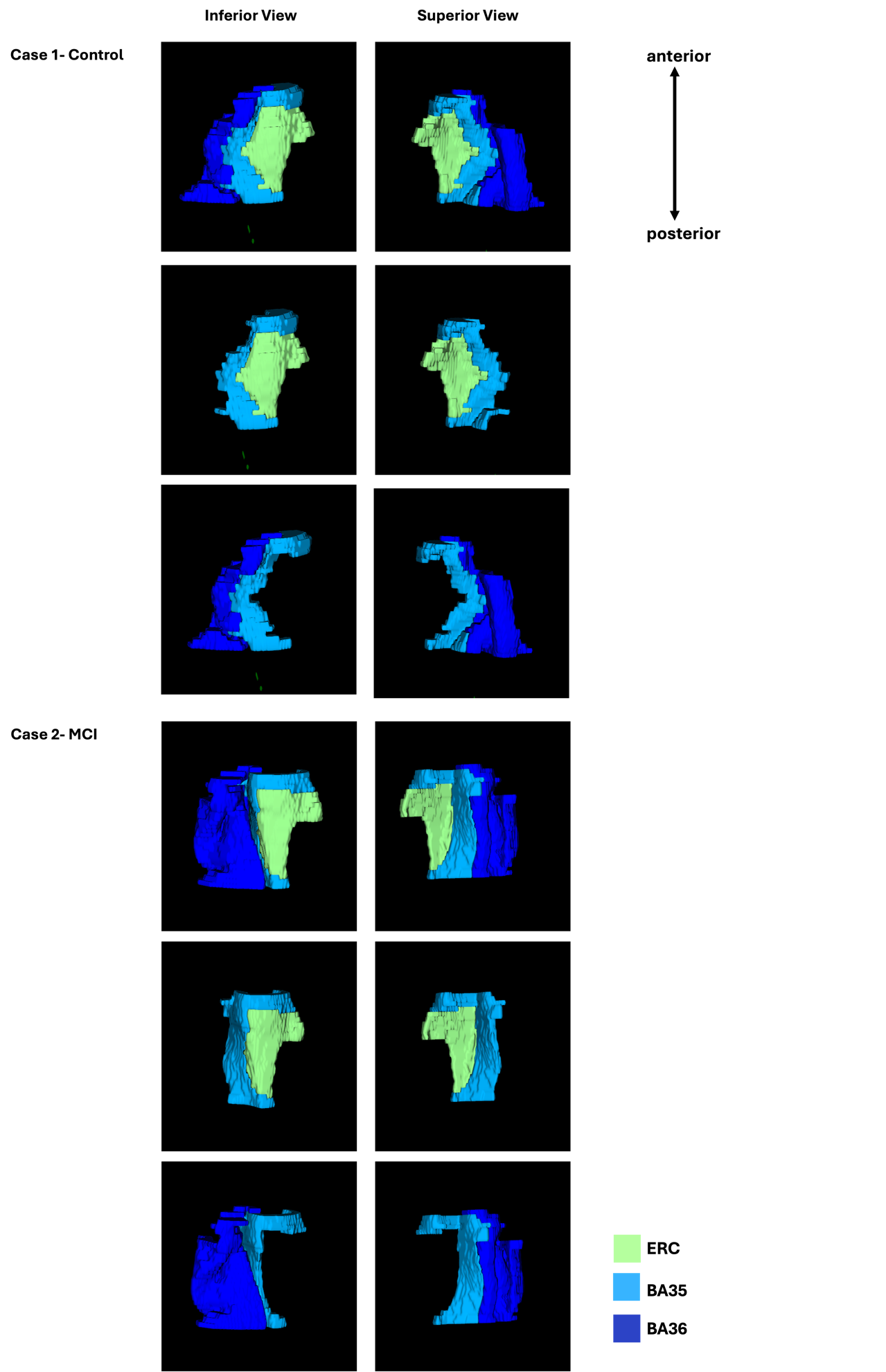


**Supplementary Figure 9.** 3D rendering of the automatic segmentation according to the developed protocol highlighting the consistency of the borders between consecutive slices in two control subjects (Cases 1 and 2) and two individuals with mild cognitive impairment (MCI; Cases 3 and 4) in the ASHS atlas set. Case 1 corresponds to the control subject shown in Supplementary Figure 5, and Case 3 corresponds to the MCI subject shown there.

Abbreviations: ASHS: Automated segmentation of hippocampal subfields; BA: Brodmann area; ERC: Entorhinal cortex; MCI: mild cognitive impairment.


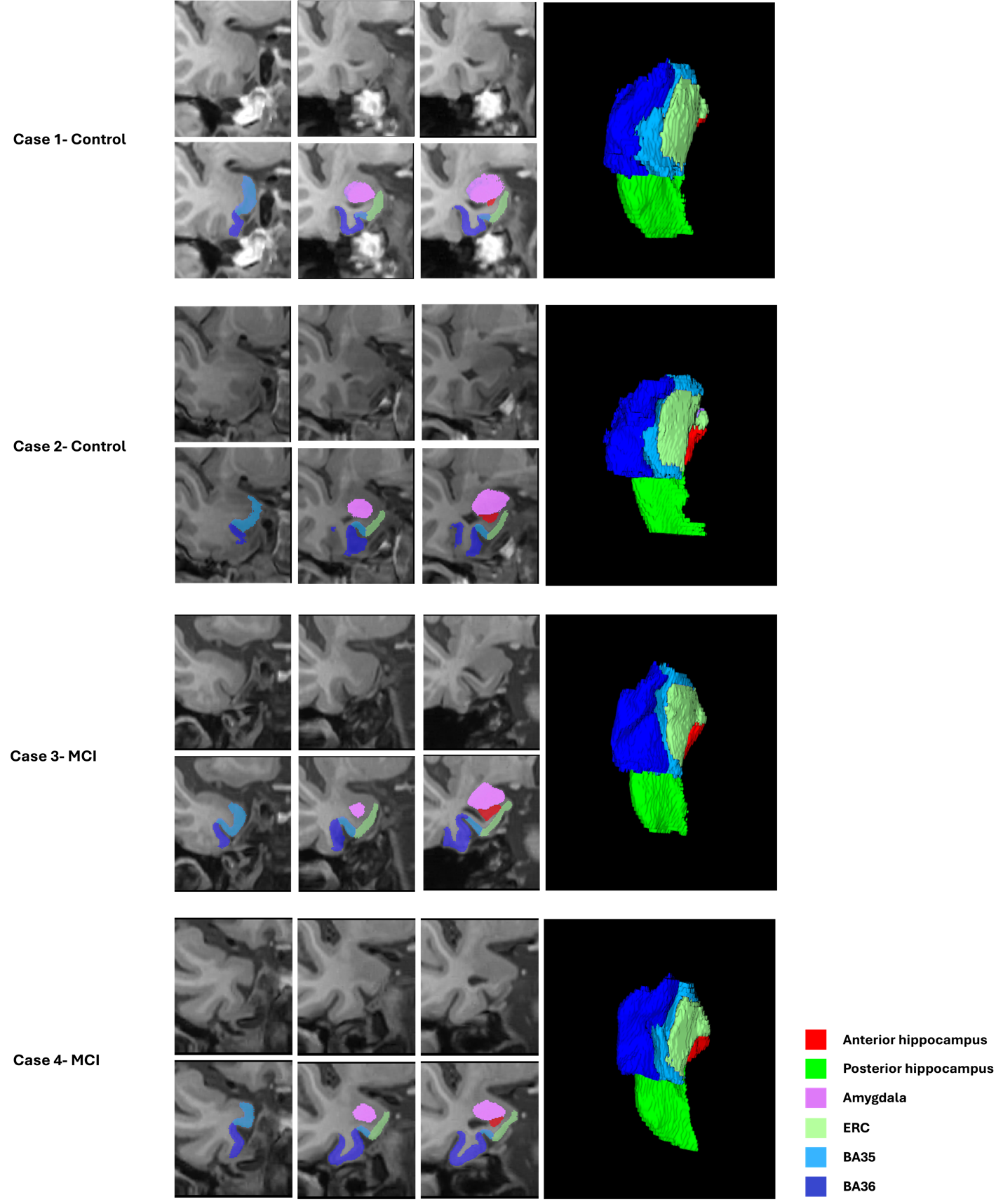


**References**

1. Braak H, Braak E. On areas of transition between entorhinal allocortex and temporal isocortex in the human brain. Normal morphology and lamina-specific pathology in Alzheimer’s disease. Acta Neuropathol (Berl). 1985 Dec 1;68(4):325–32.

2. Estudios sobre la corteza cerebral humana - University of Pennsylvania [Internet]. [cited 2024 May 8]. Available from: https://upenn.alma.exlibrisgroup.com

3. Insausti R, Muñoz-López M, Insausti AM, Artacho-Pérula E. The Human Periallocortex: Layer Pattern in Presubiculum, Parasubiculum and Entorhinal Cortex. A Review. Front Neuroanat. 2017;11:84.

4. Insausti R, Tuñón T, Sobreviela T, Insausti AM, Gonzalo LM. The human entorhinal cortex: A cytoarchitectonic analysis. J Comp Neurol. 1995;355(2):171–98.

5. Brodmann K. Vergleichende Lokalisationslehre der Grosshirnrinde in ihren Prinzipien dargestellt auf Grund des Zellenbaues [Internet]. Leipzig : Barth; 1909 [cited 2024 May 8]. 346 p. Available from: http://archive.org/details/b28062449

6. Ramo S, Cajal S. Histologie du Systeme Nerveux de l’Homme et des Verte\’ bre\’ s. 1911;

7. Lorente de Nó R. Studies on the structure of the cerebral cortex. II. Continuation of the study of the ammonic system. J Für Psychol Neurol [Internet]. 1934 [cited 2024 May 8]; Available from: https://psycnet.apa.org/record/1935-01111-001

8. Braak H. Architectonics of the Human Telencephalic Cortex. Springer Science & Business Media; 2012. 157 p.

9. Krimer LS, Hyde TM, Herman MM, Saunders RC. The entorhinal cortex: an examination of cyto- and myeloarchitectonic organization in humans. Cereb Cortex N Y N 1991. 1997 Dec;7(8):722–31.

10. Insausti R, Juottonen K, Soininen H, Insausti AM, Partanen K, Vainio P, et al. MR volumetric analysis of the human entorhinal, perirhinal, and temporopolar cortices. AJNR Am J Neuroradiol. 1998 Apr;19(4):659–71.

11. Wuestefeld A, Baumeister H, Adams JN, de Flores R, Hodgetts C, Mazloum-Farzaghi N, et al. Comparison of histological delineations of medial temporal lobe cortices by four independent neuroanatomy laboratories. BioRxiv Prepr Serv Biol. 2024 Jan 3;2023.05.24.542054.

12. Mai JK, Majtanik M, Paxinos G. Atlas of the Human Brain. Academic Press; 2015. 458 p.

13. Bailey P, Bonin G von. The Isocortex of Man. University of Illinois Press; 1951. 301 p.

14. Salinas Alaman A. Estructura de la corteza perirrinal humana. Modificaciones con el envejecimiento y la enfermedad de alzheimer [Internet] [http://purl.org/dc/dcmitype/Text]. Universidad de Navarra; 1995 [cited 2024 May 8]. Available from: https://dialnet.unirioja.es/servlet/tesis?codigo=279949

15. Blaizot X, Mansilla F, Insausti AM, Constans JM, Salinas-Alamán A, Pró-Sistiaga P, et al. The Human Parahippocampal Region: I. Temporal Pole Cytoarchitectonic and MRI Correlation. Cereb Cortex. 2010 Sep 1;20(9):2198–212.

16. Insausti R. Comparative neuroanatomical parcellation of the human and nonhuman primate temporal pole. J Comp Neurol. 2013 Dec 15;521(18):4163–76.

17. Ding SL, Van Hoesen GW, Cassell MD, Poremba A. Parcellation of Human Temporal Polar Cortex: A Combined Analysis of Multiple Cytoarchitectonic, Chemoarchitectonic and Pathological Markers. J Comp Neurol. 2009 Jun 20;514(6):595–623.

18. Ding S, Van Hoesen GW. Borders, extent, and topography of human perirhinal cortex as revealed using multiple modern neuroanatomical and pathological markers. Hum Brain Mapp. 2010 Jan 15;31(9):1359–79.

19. Ding SL, Royall JJ, Sunkin SM, Ng L, Facer BAC, Lesnar P, et al. Comprehensive cellular-resolution atlas of the adult human brain. J Comp Neurol. 2016 Nov 1;524(16):3127–481.

20. Berron D, Vieweg P, Hochkeppler A, Pluta JB, Ding SL, Maass A, et al. A protocol for manual segmentation of medial temporal lobe subregions in 7   Tesla MRI. NeuroImage Clin. 2017 Jan 1;15:466–82.
